# London Hospitals—Operations by Sir Joseph Lister, Haywood Smith and Owen—Conversazione

**Published:** 1885-09

**Authors:** Chas. C. F. Gay

**Affiliations:** London


### Foreign Correspondence.

---

King's College Hospital: Sir Joseph Lister's Operations—Women's Hospital: Emmett's Operation for Lacerated Cervix—Haywood Smith's Improved Sims' Speculum—Children's Hospital: Exsection of Elbow by Owen—Conversazione.

London, July, 1885.

*Messrs. Editor:*

After eight and a half days' voyage on board the steamer "Servia," with agreeable company, your correspondent reached the great city of London. No incident of note occurred during the passage over, save that of striking a sand-bar off Sandyhook. After vain efforts of some hours by the ship's crew to dislodge the steamer, the entire resources, at last, of the vessel, aided by tug-boats sent out by the Cunard Company, were put into requisition. Nine tugs, well manned, came to our rescue, and, in twelve hours from the time of getting on the bar, we were dragged off and sent on our way rejoicing.

The first thing for a stranger to do after reaching London and obtaining lodgings, is to mount the top of an omnibus and ride up and down and through the city; this, to be followed by study of Boedeker's maps, will give one a very good knowledge of the topography of the town. London seems to me one of the worst laid-out cities that can be imagined. It consists of a vast net-work of streets, running in all directions in the most irregular manner conceivable. There is most assuredly no evidence of the genius of an Ellicott displayed in laying out this city. After spending some days at sight-seeing, attention was turned into another channel of observation. There were London surgeons to call upon, and hospitals to visit. Lister was the one surgeon, above all others, I most desired to see; fortunately, my wishes were soon to be gratified. Letters of introduction and credentials as delegate from the New York State Medical Association to any medical society in Europe, were found of advantage. Indeed, I have been fortunate, beyond all expectations, in every step of my progress thus far since arriving here. It was my good fortune to meet Sir Joseph Lister at the *Conversazione* given by the president and members of the Royal College of Physicians, and to receive an invitation for the next day to visit King's College Hospital, and to see Lister operate at 2 o'clock.

The first operation was amputation of the left mammary gland for scirrhus. There is nothing in the removal of the breast that could be attractive of itself; but I wished to gain additional knowledge of so-called Listerism, obtained from its original source, and here was my opportunity, which was improved to the fullest extent. Sir Joseph permitted me to sit or stand by his side. It is my purpose now to describe, in detail, the operation, believing an account of it will be of interest to your readers.

In operating, Lister stood upon the left side of his patient. He first thoroughly washed the entire chest, and shaved the hair from the axilla. Spray was now turned upon the patient, and I judge the solution was weak, since I was unable scarcely to detect the carbolic acid odor at any time during the performance of the operation. His first semi-circular incision was made above the gland, and this was connected at once by another incision below the gland. The gland was dissected out from below upwards; vessels secured by hæmostatic forceps. After the gland was removed, the vessels were tied by several fine catgut ligatures, cut short. When all oozing of blood had ceased, a longitudinal incision was carried up into the axilla, and the axillary glands removed; venous hemorrhage was controlled by ligatures of the same material as that applied to the arteries. Axillary glands are always removed at all the London hospitals, whether or not there be indications of malignant disease.

The wound was closed at its central portion by three silver wire sutures of good size, only partially drawing the edges of the wound in opposition. Twelve or fifteen cat-gut ligatures in all were used in closing the wound. Three drainage tubes were used; one passed up into the axilla, one at the lower angle, and another at the upper angle of the wound.

Silk protective was laid over the wound, but it being a quarter of an inch too wide to suit the operator, he cut this amount from it.

A large quantity of gauze was now laid over the chest, and over this and around the chest was placed the Mac ntosh; and the material used was so abundant as to suggest the importance of liberal hospital endowment, in order to save the institution from bankruptcy. The cost of material is, however, much less in this country than our own.

This dressing was secured by a wide cheese-cloth bandage, which enveloped the chest. The entire dressing was applied by Lister. He had an able assistant, who was an expert in the use of the hæmostatic forceps. The operation, including dressing, lasted one hour and ten minutes.

Lister allows no one to speak during an operation, nor does he speak himself. Should anyone be so ignorant of the rules and courtesies of the operating room as to speak, he has permission to leave without standing upon the order of going. I could not but admire the stillness and silence of the operating room, and could not but think what a boon it was for the poor patient who, under the necessity of a surgical operation, could have it so neatly and skillfully performed, and the dressing so nicely applied.

Lister, I am told, is sometimes irritable, but on this occasion he was extremely gentle, and only spoke once, in an almost inaudible voice, for a moment or two, after the patient had been removed. There were, perhaps, twenty students and physicians present. I could not ascertain the strength of the spray used.

The next operation was for cleft palate, but it is needless to give any account of it.

Lister showed a case of exsection of the knee. The patient had nearly or quite recovered without suppuration of the wound. I am informed by his assistant that suppuration never occurs after Lister's operations; that he is both operator and dresser, never allowing the dressing to be removed by another person; he does this himself, and therein lies the secret probably of his successful results.

At the Women's Hospital, I saw Haywood Smith perform Emmett's operation for lacerated cervix. He used "Smith's Improved Sims' Speculum." One blade is shorter than the other and truncated; it has not the scoop shape of Sims'. One of its merits consists of the facility of removal after plugging the vagina. Smith operates with great facility with the knife instead of scissors, and uses coiled silver wire and shot to secure the sutures. He uses two knives with very delicate blades, pointed, and set in the handles at an angle of about thirty-six degrees. I took such a fancy to these knives, that the instrument maker of the hospital furnished me with them, and also the coiled wire, to take home with me.

The next day, I witnessed an operation at the Children's Hospital, by Owen, of exsection of the elbow, the principal feature of which was the preservation of the triceps, and the use of the continuous suture in closing the wound. By the courtesy of a namesake — one of London's surgeons — I had the privilege of visiting St. Bartholomew's Hospital, but must reserve, for another time, any account of this large hospital and what I saw there, and also what was observed at the London Hospital, with its eight hundred free beds.

The most notable medical event of the season was the reception given by the president and members of the Royal College of Physicians. I received a card of invitation to this reception, and, of course, accepted the honor. The words "evening dress" were printed upon the card of invitation.

The guests, numbering about two hundred, were received by Sir William Jenner, who wore a dark robe, trimmed with gold lace. Behind him, standing in a semi-circle, were eight or ten physicians, presumably officers or members of the faculty, to assist at the reception.

The name being announced, Sir William steps forward two or three paces and extends his hand, and speaks a word of welcome to his guests, after which we pass into the large hall, or library, on the walls of which hang some of the famous paintings, and in the center of the hall are tables with fifty or more microscopes, and many rare anatomical specimens, and specimens of natural history. Below is stationed the band of the Royal Artillery ; in an adjoining room, a long table, spread with all the delicacies and luxuries of the season, which means, in London, everything to eat and drink—especially to drink—except ice-water.

It was at this reception that I had the good fortune to meet Lister. Many gentlemen were present whose names are well known in our own country. The floral display, in all parts of the building, exceeded anything I have ever before seen. The great hall and its approaches had the appearance of having received the potted plants and cut flowers of two or three green-houses.

Altogether, this reception exceeded anything, for elegance, it has been my pleasure to witness, and from which I returned about midnight, greatly pleased with everybody and everything.

The courtesy of London surgeons is phenomenal. I have never seen anything equal it, and have much admiration for it, and trust I have imbibed some of the spirit of it.

At all events, I shall return home with the firm resolve, that all strangers who may chance to come among us shall receive all the attention and courtesy that I may have in my power to extend to them.

Yours truly,

CHAS. C. F. GAY.

